# Assessing the effect of indoor residual spraying (IRS) on malaria morbidity in Northern Uganda: a before and after study

**DOI:** 10.1186/s12936-016-1652-4

**Published:** 2017-01-03

**Authors:** Betty Bawuba Tukei, Andy Beke, Héctor Lamadrid-Figueroa

**Affiliations:** 1 School of Health Systems and Public Health, University of Pretoria, Pretoria, South Africa; 2 Division of Reproductive Health, Research Center for Population Health, National Institute of Public Health (INSP), Cuernavaca, Mexico

**Keywords:** Indoor residual spraying (IRS), Malaria morbidity, Slide positivity rate (SPR)

## Abstract

**Background:**

Indoor residual spraying (IRS) is known to reduce malaria transmission. In northern Uganda, a high endemic area, IRS has been implemented since 2006. Limited data however, exists on the effect of IRS on the malaria burden. This study sought to assess the effect of IRS on malaria morbidity in the high intensity area of northern Uganda. Retrospective routine data from ten health facilities in three districts which had received at least five rounds of IRS in northern Uganda was analysed. The primary outcome of interest was malaria morbidity, measured by the slide positivity rate (SPR). Descriptive statistics were used to describe the malaria morbidity stratified by age and sex. The average change in the malaria morbidity, measured by the SPR was assessed according to time, measured as calendar months. A fixed-effects linear regression model was used which included a polynomial function of time and controlled for malaria seasonality and variations between districts/facilities.

**Results:**

The total out-patient department attendance in the ten health facilities for the study period was 2,779,246, of which 736,034 (26.5%) malaria cases were diagnosed with 374,826 (50.9%) cases of under 5 years and an overall SPR of 37.5%. The percentage point (p.p.) changes in SPR according to time measured as calendar months following IRS, revealed a decreasing trend in malaria morbidity in the first 3 months following each round of IRS. The highest percentage point decrease in the SPR was observed in the second month following IRS (9.5 p.p., CI −17.85 to −1.16, p = 0.026), among patients above 5 years. The SPR decline however waned by the fourth month following IRS, with an increase in the SPR of 8.4 p.p. at district level by the sixth month, p = 0.510.

**Conclusion:**

The study results show that IRS was associated with a significant reduction in malaria morbidity in northern Uganda in the first 3 months following IRS. The malaria reduction however waned by the fourth month following IRS.

**Electronic supplementary material:**

The online version of this article (doi:10.1186/s12936-016-1652-4) contains supplementary material, which is available to authorized users.

## Background

Over the past decades, the beneficial effects of IRS in malaria prevention have been reported in both high and low malaria endemic areas [1–7]. IRS contributed to the elimination or dramatic reduction of malaria in various parts of Latin America, Asia, and Europe [8].

Uganda has some of the highest recorded malaria transmission rates in Africa with an average of 1500 infectious bites per person per year in the high intensity areas and one of the world’s highest malaria incidences of 478 cases per 1000 persons per year [9, 10]. Malaria transmission in Uganda is perennial, with two peaks following the rainy seasons, April/May and September/November. In northern Uganda, the rain season is from March/April to October. Malaria is a leading cause of morbidity and mortality with an estimated 8–13 million cases per year which account for 30–50% of outpatient visits, 15–35% of hospital admissions and 9–14% of inpatients deaths, where nearly half of the deaths are in children less than 5 years of age [11].

The primary malaria control intervention strategies implemented in Uganda include diagnosis and case management with artemisinin-based combination therapy (ACT), integrated vector management (IVM) including use of long-lasting insecticide-treated nets (LLINs), and intermittent preventive therapy in pregnancy. In 2006, IRS supported by United States Agency for International Development (USAID)/President’s Malaria Initiative (PMI) was added to the intervention strategies for malaria control initially focusing in malaria prone areas in the southwest and later concentrating on the highly endemic areas in northern Uganda. After achieving a significant decline in malaria control, the USAID/PMI mandate was to gradually phase out the spray programme and thereafter conduct continuous entomological monitoring in sentinel sites in the phased-out districts. The programmatic benefits of successful IRS programmes in Africa have been documented in several studies [12–17]. In northern Uganda, selected districts received five rounds of IRS during the study period but limited data exists on the impact of IRS on malaria morbidity in this area.

## Methods

### Study setting

This study analysed data from ten health facilities located in Apac, Gulu and Kitgum districts in northern Uganda. Northern Uganda, a post-conflict area of over two decades, is characterized by several wetlands and local forest reserves making it highly endemic to malaria. Malaria transmission in northern Uganda is also perennial, with two peaks following the rainy seasons in April/May and September/November. The following health facilities were included in this study: Aduku Health Centre IV (HCIV) in Apac district; Kitgum Government Hospital (KGH), St. Joseph’s Hospital and Namokora HCIV in Kitgum district; and Lacor hospital, Gulu Independent Hospital, Gulu Regional Referral Hospital (GRRH), Military Hospital, Lalogi HCIV and Awach HCIV in Gulu district.

### Description of IRS activities

IRS was reintroduced in Uganda in 2006 after a long period of no IRS interventions since 1960. Initial IRS activities were conducted in epidemic prone districts in the south-western part of Uganda before shifting to the highly endemic regions of northern Uganda as stipulated in the WHO position statement on IRS [18]. Initial pilot spray rounds in northern Uganda used dichlorodiphenyltrichloroethane (DDT). Consistent biannual spraying in ten districts of northern Uganda commenced in November 2009, targeting over 2.7 million people with approximately 850,000 households. The study districts had received five spray rounds over the 5-year study period starting November 2009 up to the end of the study period, December 2011 (2007–2011). The study considered these spray rounds as rounds one to five. The spray rounds were conducted biannually at different times in the different districts starting with a pyrethroid insecticide (alpha-cypermethrin) before shifting to a carbamate (bendiocarb) (see Additional file 1). The first spray round was conducted starting November 2009 with Kitgum district and ending in May 2010 with Gulu district using alpha-cypermethrin insecticide. The second to fifth spray rounds occurred at approximately 4 months’ intervals starting June 2010 to December 2011 using bendiocarb. On average, the percentage coverage of households sprayed and population protected in all the five rounds was 97%.

### Data collection procedures

Uganda has an integrated Health Management Information System (HMIS) which produces aggregated monthly routine reports on various health outcomes. Data is collected by health personnel at facility level during routine patient care and aggregated on a monthly basis by the health facility records personnel. Aggregated reports are then compiled and submitted on a monthly basis to the district health office. Of the ten districts where IRS was implemented, the study team was only able to access data from three districts: Apac, Gulu and Kitgum. This study analysed HMIS data from ten health facilities in the three districts and used it to estimate the effect of IRS on malaria morbidity in these districts of northern Uganda. The ten facilities were selected based on a criterion which considered data availability and completeness. Data was considered available and complete if permission to use it was granted by district authorities and if the variables of interest had no missing values. Retrospective data of a 5-year period (2007–2011) covering five rounds of IRS were analysed. The malaria variables analysed by the study included: malaria diagnosis categorized by age and sex; and blood smear (BS) tests including total tests done and total positive tests, segregated by age. In addition, the study used the total OPD attendance categorized by age and sex to assess the malaria prevalence. Age was categorized as <5 years and >5 years. Malaria morbidity was measured by the SPR which was the primary outcome of interest.

### Statistical analysis

The malaria morbidity as measured by the SPR was evaluated and the change in the SPR in association with time, expressed as calendar months following IRS, was assessed using a linear fixed effects regression model that included polynomials of time and controlled for malaria seasonality and variations in districts and facilities. Statistical analysis included 604 observations from the ten health facilities with 60 months ranging from January 2007 to December 2011. The 60-month time period raged from 0 to 59 with January 2007 analysed as month zero and December 2011 analysed as month 59.

The primary outcome of interest was the SPR which was also analysed by age categories of less than 5 years (<5 years) and above 5 years (>5 years). The IRS exposure variable included a 6 months’ period following each round of IRS with time expressed as months after IRS. The month of spraying was considered as a baseline month and was assigned a zero while the months following the baseline month were assigned numbers 1–6 consecutively. A month was considered a spray month if spraying occurred for more than or equal to 15 days in that month. The highest span between the spray rounds was a 6-month period.

Analyses were performed both at district and facility level using two linear fixed effects regression models regressed on the SPR as the outcome variable. Fixed effects regression deals with the lack of independence induced by the repeated-measures nature of the data [19]. The calendar time variable was included in the model as a 5th-degree polynomial to control for non-linear malaria seasonality trends. Furthermore, dummy variables for facility and district were also generated and included in the model to control for any possible background differences between facility catchment areas. For modelling purposes, to avoid giving excessive weight to facilities performing a relatively low number of tests, observations were assigned a weight according to the total number of blood smear tests done at each facility. Two models were fitted of which one model included the months past variable (included as dummy variables), the 5th degree polynomial function of calendar time and the categorical variables of facility weighted by total BS tests done while the other model replaced the facility variables with the district. The percentage change in the SPR in association to the time past following IRS expressed as calendar months was assessed, otherwise following the same procedure. A p value of <0.05 was considered statistically significant and confidence intervals (CI) were set at 95%. All statistical analysis was carried out using STATA version 12.

## Results

The characteristics of the ten health facilities are presented in Table 1. The total recorded OPD attendance in all the ten facilities over the 5-year study period was 2,779,246 of which 1,652,054 (59.4%) were female and 876,584 (31.5%) were <5 years of age. Overall a total of 736,034 (26.5%) malaria cases were diagnosed of which 374,826 (50.9%) were <5 years of age. A total of 664,000 blood slide tests were done of which 244,417 (37.5) were positive. Overall, the proportion of malaria cases among OPD attendees was highest in female 57%, as compared to male 43%, and was higher in patients less than 5 years of age 51%, as compared to patients above 5 years of age, 49%.

**Table 1:** Characteristics of the study population

District	Facility	Total OPD attendance	OPD female (% of total OPD)	OPD <5 years (% of total OPD)	Malaria diagnosis (% of total OPD)	Malaria diagnosis <5 years (% of total diagnosis)	Total BS tests done	Positive BS (SPR)
Apac	Aduku HCIV	150,090	90,625 (60.4)	51,249 (34.1)	45,120 (30.1)	25,208 (55.9)	58,884	23,362 (39.7)
Gulu	Lacor Hospital	845,254	515,362 (61.0)	286,836 (33.9)	164,117 (19.4)	110,229 (67.2)	289,288	95,207 (32.9)
	Military Hospital	54,399	22,343 (41.1)	13,330 (24.5)	18,331 (33.7)	5000 (27.3)	11,347	2981 (26.3)
	GRRH	246,216	152,137 (61.8)	48,654 (19.8)	73,620 (29.9)	25,406 (34.5)	24,698	8539 (34.6)
	Lalogi HCIV	142,211	85,412 (60.1)	52,419 (36.9)	36,032 (25.3)	18,784 (52.1)	26,092	15,299 (58.6)
	Gulu Independent	44,130	23,557 (53.4)	6382 (14.5)	4969 (11.3)	1574 (31.7)	16,802	2640 (15.7)
	Awach HCIV	86,815	56,469 (65.0)	27,615 (31.8)	35,123 (40.5)	15,468 (44.0)	7462	2264 (30.3)
Kitgum	KGH	387,499	226,537 (58.5)	114,172 (29.5)	126,286 (32.6)	58,166 (46.1)	91,442	38,229 (41.8)
	SJH	187,745	105,085 (56.0)	71,644 (38.2)	65,346 (34.8)	37,771 (57.8)	75,915	30,097 (39.6)
	Namokora HCIV	173,438	93,578 (54.0)	58,417 (33.7)	45,483 (26.2)	20,697 (45.5)	5857	2798 (47.8)
Total		2,779,246	1,652,054 (59.4%)	876,584 (31.5%)	736,034 (26.5%)	374,826 (50.9%)	664,000	244,417 (37.5)

*OPD* Out patients Department, *BS* blood slide, *SPR* slide positivity rate, *HCIV* Health Centre IV, *GRRH* Gulu Regional Referral Hospital, *KGH* Kitgum Government Hospital, *SJH* St. Joseph’ Hospital

### Changes in the SPR as a function of time after IRS

Considering the baseline time, the month of spraying, in relation to the months following IRS, there was a sustained decrease in the SPR adjusted for variations at both facility and district level in the first 3 months following IRS. In the second month following IRS, a statistically significant decrease in the SPR of 6.0 percentage point (p.p.) and 6.5 p.p. was observed adjusted for variations at both facility and district level (CI −11.73 to −0.36, p = 0.037 and CI −12.56 to −0.41, p = 0.037, respectively). Similarly, a continuous decrease was observed when the SPR was analysed by age categories both for patients less than 5 years of age and those above 5 years (CI −17.85 to −1.16, p = 0.026 and CI −8.03 to 5.55, p = 0.718), respectively in the second month following IRS. However, the decreasing trend in the SPR started to reverse to an increase in the fourth to sixth months following IRS. In the sixth month following IRS, an increase of 8.4 p.p. and 5.3 p.p. was observed after adjustment for variations at both facility and district level respectively (CI −16.66 to 33.39, p = 0.510 and CI −18.04 to 28.58, p = 0.655 respectively). Analysis of the SPR by age also revealed an increase among patients less than 5 years of age and among patients above 5 years of age.

The details of the regression results 6 months after IRS adjusted for variations at facility level including analysis by age categories are illustrated in Table 2. The regression results adjusted for variations at district level are given in Table 3, which shows 8.4 p.p. increase in the SPR by the sixth month following IRS.

**Table 2:** Regression results adjusted for variations at facility level

Timing in relation to IRS	p.p. change	p value	95% confidence interval	
			Lower boundary	Upper boundary
Percentage change in total SPR				
1 month after IRS	−3.45	0.208	−8.86	1.95
2 month after IRS	−6.04	0.037	−11.73	−0.36
3 month after IRS	−6.53	0.040	−12.74	−0.31
4 month after IRS	−0.17	0.958	−6.53	6.19
5 month after IRS	−2.74	0.461	−10.05	4.58
6 month after IRS	5.27	0.655	−18.04	28.58
Percentage change in SPR <5 years				
1 month after IRS	−2.19	0.503	−8.64	4.27
2 month after IRS	−1.24	0.718	−8.03	5.55
3 month after IRS	−3.36	0.372	−10.77	4.06
4 month after IRS	2.78	0.470	−4.80	10.35
5 month after IRS	−1.34	0.764	−10.13	7.45
6 month after IRS	0.76	0.960	−29.37	30.89
Percentage change in SPR >5 years				
1 month after IRS	−4.03	0.317	−11.95	3.90
2 month after IRS	−9.50	0.026	−17.85	−1.17
3 month after IRS	−8.18	0.079	−17.31	0.95
4 month after IRS	0.73	0.876	−8.58	10.04
5 month after IRS	−3.40	0.533	−14.16	7.36
6 month after IRS	7.95	0.647	−26.25	42.14

*p.p change* percentage point change

**Table 3:** Regression results showing percentage point change in total SPR adjusted for variations at district level

Timing in relation to IRS	Percentage point change	p value	95% confidence interval	
			Lower boundary	Upper boundary
1 month after IRS	−4.18	0.157	−9.98	1.63
2 month after IRS	−6.48	0.037	−12.55	−0.41
3 month after IRS	−7.11	0.038	−13.80	−0.41
4 month after IRS	−0.39	0.912	−7.27	6.50
5 month after IRS	−2.97	0.459	−10.87	4.93
6 month after IRS	8.37	0.510	−16.66	33.39

Two linear fixed effects regression models were regressed on the SPR as the outcome variable both at district and hospital level. The models are shown below

*Hospital model* SPR = β_0_ + β_1_ (time) + β_2_ (months past after spraying) + β_3_ (Hospital)

*District model* SPR = β_0_ + β_1_ (time) + β_2_ (months past after spraying) + β_3_ (District)

The regression results adjusted for variations at district level and the adjusted confidence intervals of the percentage changes in the SPR 6 months after IRS reveal the same results with a decrease in the SPR 1–3 months after IRS which wanes out in the fourth month following IRS. The same results are obtained when the SPR is analysed by age category as shown in Table 6. The SPR increases by the sixth month when compared to the spray month, the reference month = zero

### SPR trends after IRS

**Fig. 1:**
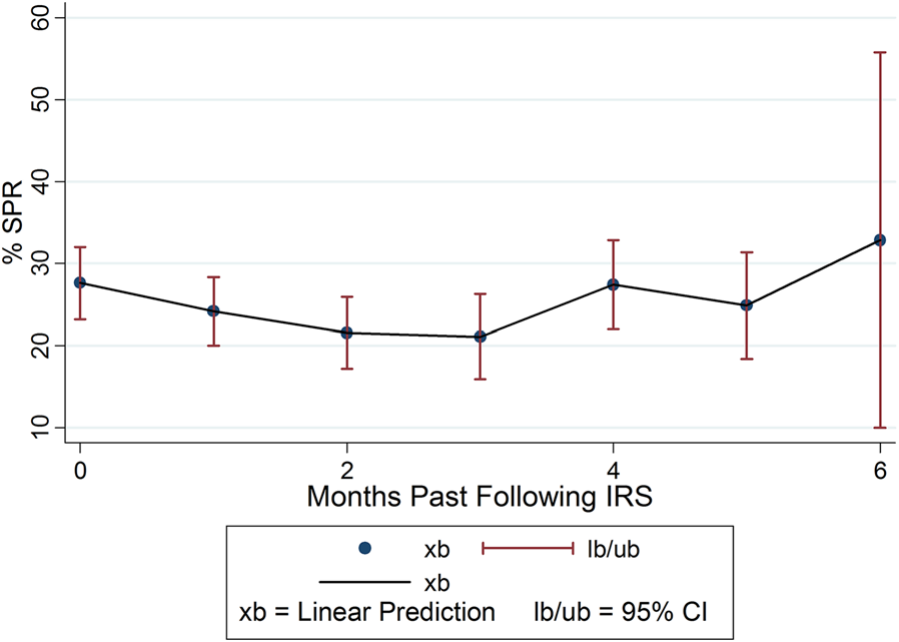
Trend of changes in the SPR in the months following IRS

**Fig. 2:**
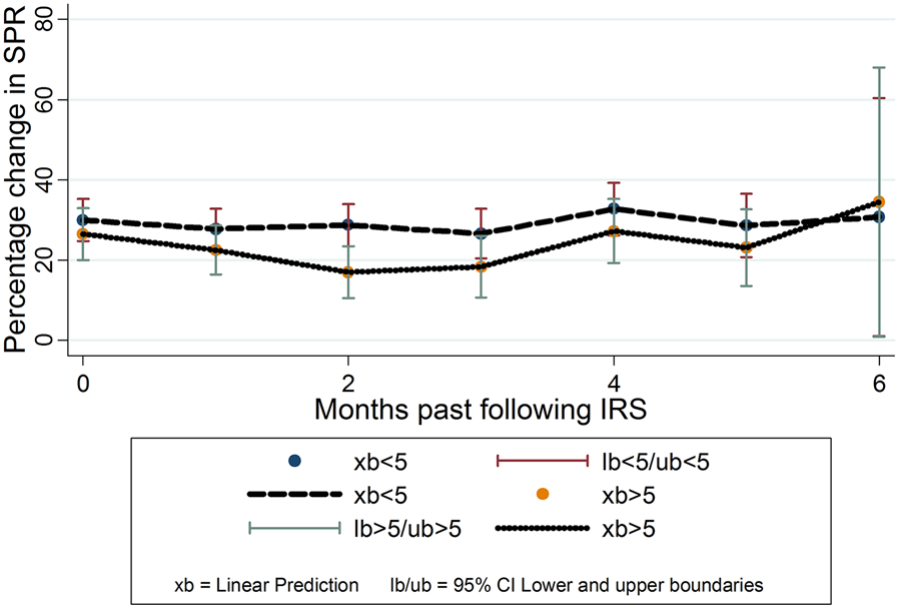
Change in the SPR over time by age category

The SPR had a declining trend for the first 3 months following IRS which however waned by the fourth month following IRS as illustrated in Fig. 1. A similar declining trend which waned off by the fourth month was observed when the SPR was analysed by age category. The SPR was higher among patients less than 5 years of age as compared to those above 5 years of age as illustrated in Fig. 2.

The annual SPR at facility level confirms a decline in the SPR in the years following IRS, 2010 and 2011 at all the ten facilities. In Lacor hospital for example, the annual SPR declined to 6% in 2011 from 41% in 2009. The total annual SPR for the ten health facilities over the study period is highlighted in Table 4. The adjusted confidence intervals of the percentage point changes in the SPR at facility level over time are in Table 5.

**Table 4:** Annual SPR at facility level across the study period

Hospital	SPR 2007	SPR 2008	SPR 2009	SPR 2010	SPR 2011
Aduku	62.60	35.29	43.96	41.65	22.32
St. Joseph’s	42.87	44.34	56.37	33.92	6.74
KGH	48.69	43.19	43.59	41.55	20.16
Lacor	34.29	26.40	41.15	36.32	6.06
Military	39.22	20.12	31.00	24.48	16.48
Namokora HCIV	32.59	68.46	72.32	42.87	23.58
Gulu Independent	11.71	10.47	34.63	20.48	6.98
Lalogi HCIV	65.36	57.47	67.43	52.72	31.05
Awach HCIV	21.92	25.64	46.02	33.56	6.79
GRRH	26.91	30.43	39.93	35.19	21.70

*KGH* Kitgum Government Hospital, *GRRH* Gulu Regional Referral Hospital

**Table 5:** Adjusted confidence intervals of the percentage point changes in the SPR at facility level

Months past after IRS	xb	lb	ub
Overall SPR			
0	27.61	23.20	32.02
1	24.16	20.00	28.31
2	21.57	17.18	25.95
3	21.08	15.89	26.28
4	27.44	21.99	32.88
5	24.87	18.38	31.37
6	32.88	10.00	55.76
SPR <5 years			
0	29.97	24.70	35.25
1	27.79	22.81	32.76
2	28.73	23.47	33.99
3	26.62	20.41	32.82
4	32.75	26.26	39.24
5	28.64	20.78	36.50
6	30.74	1.049	60.42
SPR >5 years			
0	26.53	20.05	33.00
1	22.50	16.41	28.60
2	17.02	10.57	23.48
3	18.35	10.70	26.00
4	27.26	19.30	35.23
5	23.13	13.54	32.71
6	34.47	0.92	68.02

*xb* linear prediction, *lb* 95% confidence interval, lower boundary, *ub* 95% confidence interval, upper boundary

**Fig. 3:**
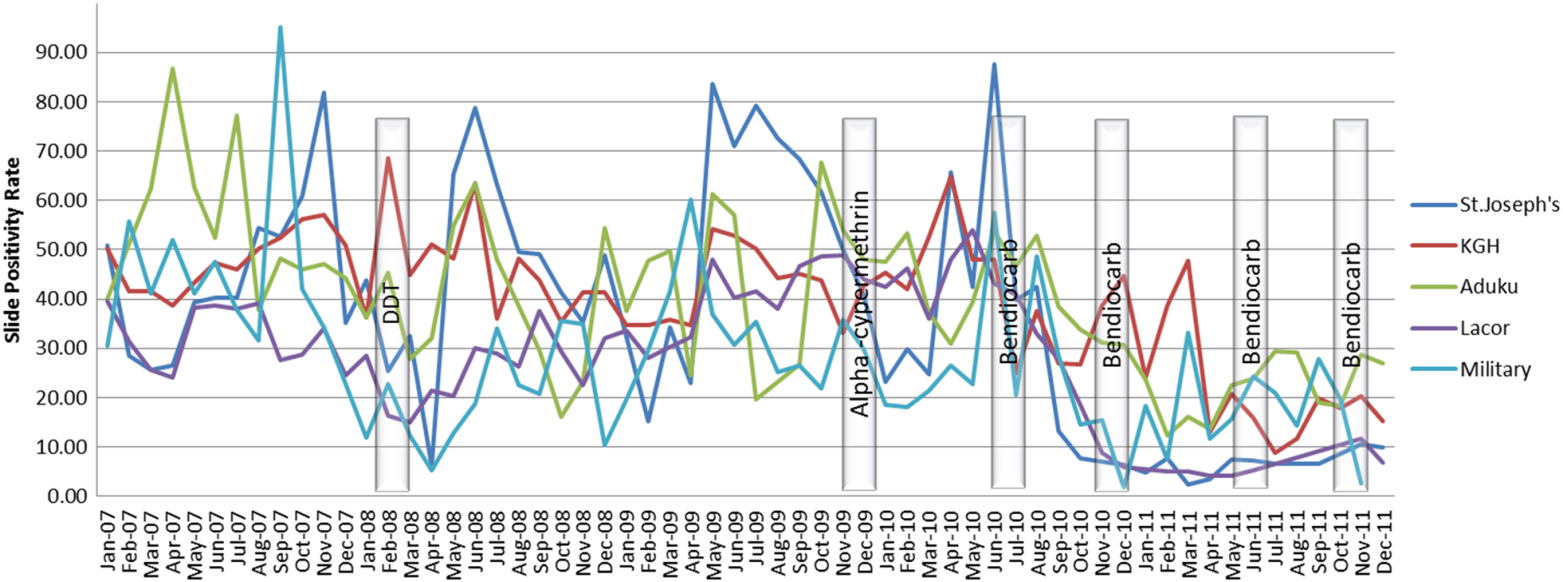
Monthly trends in the SPR in five health facilities

**Fig. 4:**
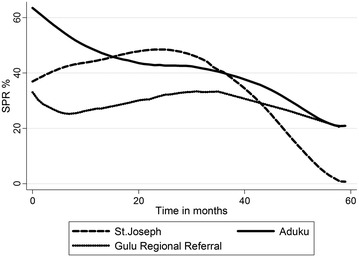
Monthly change in SPR for Aduku, Gulu regional referral and St. Joseph. There was a declining trend in the SPR in the months following IRS across the 60 months study period, which shows a declining trend in the SPR percentage points from three facilities; one from each district

**Fig. 5:**
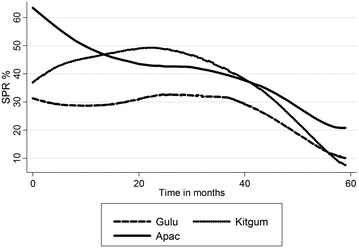
Monthly percentage point change in SPR for the three study districts over the study period. The figure illustrates the trend in the SPR percentage point in the 60 months’ study period at district level. A declining trend was observed in the SPR percentage points in all the three districts

There was a notable decline in the monthly SPR across the ten facilities in the months following IRS. The monthly trend in the SPR from five health facilities across the study period is illustrated in Fig. 3. The percentage point differences in the SPR between districts with Apac district as the reference district and the percentage point differences in the SPR between facilities with Aduku HCIV as the reference facility are highlighted in Table 6. The monthly SPR percentage points for Aduku HCIV, Lacor hospital, St. Joseph’s Hospital across the 60 months of the study period is also given on Fig. 4. The trend in the SPR percentage points at district level across the study period is given on Fig. 5. Both figures illustrate a declining trend in the SPR percentage points in the months following IRS.

**Table 6:** Percentage point change in the SPR adjusted for variations at district and facility level following IRS

	Change	p value	95% confidence interval	
			Lower boundary	Upper boundary
Percentage point change in the SPR at district level				
District				
Gulu	−8.59	0.006	−14.62	−2.56
Kitgum	−4.42	0.083	−9.42	0.58
Percentage point change in the SPR at facility level				
Facility				
Awach HCIV	−10.47	0.032	−20.00	−0.94
GRRH	−3.48	0.620	−17.35	10.38
Gulu Independent	−22.02	0.000	−33.90	−10.13
Kitgum Hospital	−0.88	0.734	−6.02	4.25
Lacor	−10.16	0.062	−20.83	0.51
Lalogi HCIV	6.04	0.235	−3.97	16.05
Military Hospital	−11.42	0.015	−20.53	−2.28
Namokora HCIV	2.22	0.682	−8.48	12.93
St. Joseph	−9.35	0.001	−14.59	−4.11

This table highlights the percentage point differences in the SPR between districts with Apac district as the reference district and the percentage point differences in the SPR between facilities with Aduku HCIV as the reference facility. In general, the percentage point changes in the SPR adjusted for variations at facility and district level show a declining trend as compared to the reference points

## Discussion

In this study, IRS was associated with a significant decrease ranging from 2.2 to 9 p.p. in malaria morbidity as measured by the SPR in northern Uganda following five rounds of spray with alpha-cypermethrin and bendiocarb. The malaria morbidity decrease was more pronounced in the second month after IRS though the effect waned by the fourth to sixth month after IRS.

IRS, mainly with DDT, was the principal method by which malaria was eradicated or greatly reduced in many countries in the world between the 1940s and 1960s [8]. Reduction in malaria morbidity following IRS has been reported in similar studies conducted in various parts of Africa. A recent study on the effect of IRS on malaria and anaemia in northern Uganda associated IRS to a reduction in the malaria burden in high transmission settings. The study revealed parasitaemia prevalence among children <5 years of age was lower in the two IRS districts (Apac and Pader) when compared with the non-IRS district (Lira): 37.0% and 16.7 versus 49.8%, p < 0.001. Anaemia prevalence was also significantly lower in the two IRS districts: 38.8% and 36.8 versus 53.0%, p < 0.001. [20]. The results obtained in this study similarly show a decrease in malaria morbidity as measured by the SPR following IRS. In this study, IRS was associated with a 3.5 p.p. to 7.1 p.p. decrease in the SPR in the first 3 months following IRS. The decrease ranging from 2.2 p.p. to 9 p.p. was also observed in the SPR when analysed by age category.

Another retrospective descriptive study to analyse the changes in the malaria morbidity and mortality and assess whether IRS had an impact on the malaria trends, conducted in Mpumalanga Province, South Africa, revealed a significant decrease in the incidence of malaria following IRS from 385 in 2001 to 2002 to 50 cases per 100,000 population in 2008 to 2009 (p < 0.005) [21]. Similarly, in this study, a decreasing trend in malaria morbidity as measured by the SPR was observed in the first 3 months following IRS. In this study however, the decline waned in the fourth to sixth month following IRS.

A similar before and after IRS study conducted to assess the impact of IRS on malaria morbidity in western Uganda, Kabale district in 2009 found that there was a large decrease in the SPR in the first 4 months after IRS both for patients <5 (47 versus 14%, p < 0.001) and >5 (26 versus 9%, p < 0.001) years of age, but this effect waned over the subsequent 12 months [22]. Likewise, in this study, there was a significant decrease in the SPR both among patients <5 and those >5 in the first 3 months after IRS. However, this effect waned over the subsequent 3 months and an increase in the SPR started to be observed up to the sixth month after IRS.

Different risk factors for malaria transmission in Africa have been identified by various studies including environmental factors such as altitude, temperature and climate; biological factors like the anopheles vector density; and human related factors and activities such as socio-economic status, health access, migration, gender, age, availability of control activities like IRS and LLITN, housing structure and land use [23–25] This study did not assess all of these factors; however, it is in agreement with other studies that showed that females and children are the most vulnerable to the risk of malaria [26]. Generally, the highest malaria prevalence occurs in children and females and the positive malaria diagnosis rate decreases with age. In this study, the malaria prevalence among OPD attendees was highest in female as compared to male and was higher in patients less than 5 years of age as compared to patients older than 5 years of age.

A study conducted in a high-transmission-intensity area of Northern Uganda that assessed the association between IRS and malaria morbidity, revealed a much greater decrease in the odds of malaria in patients less than 5 years of age following three rounds of IRS with bendiocarb (ORs 0.34, 0.16, 0.17 respectively, p < 0.001 for all comparisons) [27]. In this study however, the protection by IRS was more pronounced in patients greater than 5 years of age, up to 9 p.p. decrease. However, a notable percentage point decrease of 3 p.p. was observed in patients less than 5 years of age following IRS. Evidence from studies is suggestive of an age-acquired malaria immunity which makes younger patients more vulnerable to the disease [28]. Younger patients are therefore more dependent on malaria control interventions like IRS to prevent malaria transmission. The results of this study provide further evidence of the effectiveness of IRS in providing protection from malaria transmission in both age categories. As suggested by other studies, several years of IRS may be needed in very high transmission areas before a significant health impact on malaria morbidity can be achieved [29].

In Uganda, IRS is now recognized as one of the malaria control interventions in the country. IRS campaigns were started in Uganda in 2006 with support from USAID/PMI. Since 2009, the IRS activities were concentrated in ten high transmission districts in northern Uganda covering over 2.7 million people and 850,000 households. However; in 2014, the spray programme was phased out from these districts and emphasis was diverted to another 14 districts in Northern and Eastern Uganda. Following this decision, an increase in malaria cases was reported in all the ten districts [30]. These resurgence of malaria outbreaks in the region clearly demonstrates the beneficial effect of IRS in these districts.

Withdrawal of IRS requires a robust transition strategy which includes the application of IVM principles including use of LLINs; robust epidemiological and entomological monitoring, including setting up sentinel sites for malaria surveillance, thereby strengthening district capacity to diagnose malaria by laboratory and clinical diagnosis; and regular communication of entomological vector population data with relevant implementing partners in order to promptly manage the clinical cases of malaria that may arise.

## Study limitations

This study lacked a control group to better quantify the effect of IRS in comparison with non IRS districts, which prompts us to be cautious about making causal inferences on the estimated effect of the IRS. However, the overall decline in SPR in the months following the spraying as well as the subsequent waning of the effect, point to a causal relationship between both variables, as the study team is not aware of any other simultaneous phenomenon or intervention that could explain this behaviour besides the mass distribution of LLITNs in the study area. The mass distribution of LLITNs could unlikely explain the findings as similar studies that conducted separate analyses for IRS and LLITNs showed that ITNs were not associated with a significant reduction in malaria prevalence while IRS provided a significant added benefit in malaria reduction even in settings were ITN ownership is high. Although natural seasonal variations in SPR do exist, this is unlikely to explain the findings as the sprayings did not occur simultaneously in all districts and the inclusion of calendar time variable in the models controlled for the non-linear malaria seasonality trends. A larger sample size would have enabled an easier detection of significant differences in the changes in the SPR over time, although the study still was able to find a significant effect of IRS. Despite the study limitations, the results presented draw attention to the effect of IRS on malaria morbidity, suggesting the reduction of malaria morbidity and/or even elimination are possible following a sustained and well managed IRS programme.

## Conclusion

The results of this study show that an effective IRS programme was associated with a reduction in malaria morbidity. In this study, malaria morbidity in northern Uganda, as measured by the SPR, was reduced in the first 3 months following IRS. The decreasing effect of the SPR however waned 4–6 months following IRS. Further studies are needed to expound the waning effect and to assess whether a maximum of 3 months’ interval in the spray rounds would retain a decreasing trend in the SPR. Furthermore, studies to assess the efficacy of different classes of insecticides and possible insecticide resistance in the spray area should also be conducted.

This study recommends an up-scaling of a sustained and well-managed IRS programme across the nation with a special focus on malaria high transmission intensity areas. Furthermore, the study recommends an enforcement of a robust transition strategy following IRS withdrawal. Additionally, this study recommends conducting further studies to better quantify the impact of IRS with the use of a control group.

## Additional file

**Additional file 1.** Details of indoor residual spraying rounds.

## Abbreviations

BS: blood slide
CI: confidence interval
DDT: dichlorodiphenyltrichloroethane
HMIS: Health Management Information System
IRS: indoor residual spraying
LLIN: long-lasting insecticide-treated net
OPD: Out Patients Department
p.p.: percentage point
SPR: slide positivity rate
