# Supplementary material for: Assessing the effect of indoor residual spraying (IRS) on malaria morbidity in Northern Uganda: a before and after study
Source: Malar J. 2017 Jan 3;16:4. doi: 10.1186/s12936-016-1652-4 (PMC5209922; doi:10.1186/s12936-016-1652-4)
Supplement: Supplementary file 1 — Additional file 1. Details of indoor residual spraying rounds. [file 12936_2016_1652_MOESM1_ESM.docx]

## Additional file 1

**Details of Indoor Residual Spraying Rounds**

| **Spray Round (Insecticide Used)** | **District** | **Spray Dates** | **Households Sprayed (%)*** | **Population Protected (%)*** |
| --- | --- | --- | --- | --- |
| 1^st^ (Alpha-cypermethrin ) | Kitgum | 4th Nov-23rd Dec 2009 | 97.0 | 97.5 |
|  | Apac | 23rd Feb-31st Mar 2010 | 99.9 | 99.9 |
|  | Gulu | 5th April-14th May 2010 | 97.6 | 98.0 |
| 2^nd^ (Bendiocarb ) | Kitgum | 11th June-2nd Aug 2010 | 97.8 | 97.8 |
|  | Apac | 23rd Aug-21st Sept 2010 | 99.6 | 99.6 |
|  | Gulu | 6th Sept-30th Sept 2010 | 98.5 | 99.0 |
| 3^rd^ (Bendiocarb ) | Kitgum | 3rd Nov-10th Dec 2010 | 98.9 | 99 |
|  | Apac | 5th Jan-30th Jan 2011 | 99.4 | 99.3 |
|  | Gulu | 9th Mar-31st Mar 2011 | 98.3 | 98.7 |
| 4^th^ (Bendiocarb ) | Kitgum | 9th May-4th June 2011 | 94.7 | 93.3 |
|  | Apac | 23rd May-20th June 2011 | 96.8 | 96.8 |
|  | Gulu | 13th Oct-13th Nov 2011 | 95.4 | 96.3 |
| 5^th^ (Bendiocarb ) | Kitgum | 26th Oct-24th Nov 2011 | 94.2 | 92.3 |
|  | Apac | 9th Nov-10th Dec 2011 | 93.1 | 94.0 |

*Figures from the Uganda IRS Project Annual reports

Spraying was conducted at different times in the three districts starting November 2009 to December 2011 across the study period. A total of five spray rounds were completed in this period. The actual dates when the spraying occurred in the different districts are illustrated in the table.
